# Quantification of lettuce leaf DUS test traits and phenotypic fingerprint construction for variety identification

**DOI:** 10.1016/j.plaphe.2026.100197

**Published:** 2026-03-06

**Authors:** Guangjie Qiu, Weiliang Wen, Xiaoqian Chen, Chuanyu Wang, Si Yang, Xinyu Guo, Chunjiang Zhao

**Affiliations:** a Institute for the Smart Agriculture, Jilin Agricultural University, Changchun, 130118, China; b Beijing Key Laboratory of Digital Plant, National Engineering Research Center for Information Technology in Agriculture, Beijing, 100097, China; c Information Technology Research Center, Beijing Academy of Agriculture and Forestry Sciences, Beijing, 100097, China

**Keywords:** Lettuce leaf, DUS test traits, Phenotypic fingerprint, High-throughput phenotyping, Intelligent breeding

## Abstract

Rapid and accurate identification of DUS (Distinctness, Uniformity, and Stability) test traits in lettuce leaves is essential for advancing multi-omics-driven intelligent breeding. It also plays a critical role in germplasm protection and enhancing agricultural competitiveness. However, the phenotypic traits of lettuce leaves are highly diverse and complex due to both genotypic variation and environmental influences, posing significant challenges for precise DUS trait quantification. To address these challenges, we propose a high-precision phenotypic trait extraction pipeline and introduce an interpretable phenotypic fingerprinting framework for lettuce subgroup identification. First, a lightweight semantic segmentation network guided by group attention is developed to extract leaf components. Then, shape, color, and texture traits are comprehensively quantified. Following UPOV (International Union for the Protection of New Varieties of Plants) guidelines, we establish quantitative methods for seven DUS test traits: leaf shape, leaf tip shape, leaf margin shape, leaf vein shape, color hue, brightness, and anthocyanin coloration. Finally, PCA (Principal component analysis) was used to select 13 key traits, capturing over 95.82% of the total variance, for constructing “phenotypic ID” of lettuce varieties. Experiments conducted on 709 lettuce leaf image datasets showed that the accuracy of subgroup identification based on phenotypic fingerprints reached 98.59%. This study offers a scalable approach for automated DUS test trait evaluation and intelligent crop variety identification, providing a novel paradigm with strong potential for application in precision breeding and germplasm resource management.

## 1 Introduction

Lettuce (*Lactuca sativa L.*) is an annual or biennial herb in the Asteraceae family, widely cultivated across the globe. As a major leafy vegetable, it provides essential nutrients and holds considerable economic value [1,2]. Accurate evaluation of phenotypic traits is critical for improving yield, quality, and breeding efficiency [3,4].

The importance of plant phenotypes has been recognized by agronomists and researchers since ancient times. Phenotypic traits have long been selected to enhance crop yield and quality, making them among the oldest traits studied in agricultural science [[5], [6], [7]]. Plant phenotypes are shaped by both genetic and environmental factors, and breeders commonly leverage this variation to achieve specific breeding objectives [8,9]. Quantitative characterization of phenotypic traits is therefore essential for crop improvement and for uncovering the mechanisms underlying key biological traits. A major challenge in plant phenotyping research is the rapid acquisition of sufficient, high-quality phenotypic data [10]. Traditional morphology-based approaches can capture observable traits such as leaf color and leaf shape, but these methods are often subjective and limited in scale [11]. Chemical analysis methods, though accurate and reliable, are time-consuming, costly, and destructive [12]. With recent advances in agricultural informatization, artificial intelligence, and machine learning, new image-based phenotyping strategies have emerged [13]. As an integral component of modern plant phenomics, these technologies quantify morphological, physiological, and biochemical responses to environmental stresses, supporting yield improvement, quality enhancement, and resource-efficient crop management [14,15].

Rapid advances in plant phenomics over the past decade have enabled the analysis of phenotypes using two-dimensional (2D) and three-dimensional (3D) imaging techniques [[16], [17], [18]]. These technologies include digital cameras, scanners, magnetic resonance imaging (MRI), computed tomography (CT), spectroscopy, and radar-based point cloud systems. High-throughput plant phenotyping platforms leverage these imaging tools to quantitatively assess genotype–phenotype relationships. 2D and 3D imaging primarily capture external morphology and growth dynamics of plants [19,20], whereas spectral imaging techniques provide complementary insights into internal physiological status and biochemical properties [21,22].

To this end, image-based phenotyping provides an automated, non-destructive, and cost-effective approach for quantifying lettuce leaf traits [23]. Interpretable and highly distinguishable phenotypic characteristics across large lettuce populations are essential for phenotypic identification and for analyzing phenotype–gene–environment associations. Detailed structural phenotypes of lettuce leaves offer valuable cues for taxonomic characterization and supply an accurate computational basis for functional leaf analysis. Morphologically, lettuce leaves can be divided into distinct functional regions [24]. For example, leaf surface area reflects the plant's photosynthetic capacity and can be used to predict plant development and fruit quality. Leaf size also informs cultivation practices, including plant training, pruning, irrigation, and nutrient management, in addition to influencing photosynthesis and transpiration. The petiole reflects the plant's dormancy state, with shorter petioles produced under dormant conditions. Crown diameter and girth are closely related to plant biomass and growth, to some extent.

One of the essential procedures before a crop variety can be accepted for production is the DUS test [25]. The purpose of the DUS test is to identify trait variation within species, ensure the stability of traits within varieties, assess the consistency of phenotypic traits, and examine the uniqueness of internal variation. As a phenotype-centered assessment grounded in morphological observation, the DUS test plays a critical role in variety identification and registration, yet it remains a labor-intensive and technically demanding process [26]. In current practice, DUS evaluations are conducted manually. This approach faces several limitations: the large number of traits to be assessed increases the risk of human error, subjective judgement can lead to inconsistency among evaluators, and manual measurements require substantial time and labor investment [27]. With the advancement of computer vision and digital agriculture, automated morphological measurement has emerged as a promising alternative. Image-based approaches improve trait recognition accuracy, reduce evaluation time, and enhance repeatability and objectivity, thereby addressing many of the challenges inherent to traditional manual DUS testing.

Phenotypic fingerprinting has emerged as a promising strategy for crop variety identification. By quantitatively extracting morphological, color, and texture traits, it generates a unique and stable “identity” for each variety, providing a precise basis for germplasm identification, resource conservation, and breeding applications. However, phenotypic fingerprinting remains in an early developmental stage and lacks a unified, mature analytical framework [28]. Existing studies focus mainly on morphological feature extraction and classification [29], while issues such as inadequate data standardization, limited high-throughput processing capacity, and insufficient interpretability are yet to be fully resolved. Notably, research in related domains particularly face recognition has achieved substantial advancements in robust feature encoding and identity verification. Deep learning models such as convolutional neural networks (CNNs) and generative adversarial networks (GANs) demonstrate strong capabilities in structured feature extraction under complex imaging conditions [30,31], and feature-point encoding and matching techniques [32] provide methodological inspiration for constructing phenotypic fingerprints for crop variety identification.

Phenotypic characterization is a critical component of DUS testing. Accurately capturing phenotypic trait variation in new varieties under defined environmental conditions provides essential technical support for reliable DUS assessments. In this study, high-throughput phenotyping techniques were employed to construct phenotypic fingerprints from a morphological and histological perspective, thereby enhancing standardization and improving the efficiency of variety identification. Using lettuce as the research subject, this work addresses the pressing need for high-throughput and interpretable phenotypic identification within the DUS testing process. By integrating computer vision methods, we established a streamlined phenotypic analysis pipeline capable of accurately quantifying leaf shape, color, and texture traits under controlled conditions. On this basis, standardized phenotypic fingerprints were generated to support subgroup variety identification. The goal of this study is to promote innovation in lettuce germplasm characterization, provide a scientific foundation for assisted breeding, and offer methodological references for advancing DUS testing. The main contributions of this study are summarized as follows:

A high-throughput pipeline was developed for lettuce leaf phenotypic trait extraction, enabling automatic segmentation of multi-component leaf structures and automated extraction of phenotypic traits. This pipeline provides technical support and a feasible approach for the efficient acquisition and standardized expression of large-scale and diverse lettuce phenotypic information.

A high-precision quantification method for lettuce leaf DUS test traits based on UPOV guidelines was proposed, comprehensively quantifying key traits such as leaf shape, leaf tip shape, leaf margin shape, leaf vein shape, color hue, color brightness, and anthocyanin coloration. This method offers a reliable basis for the objective, consistent, and efficient evaluation of DUS test traits.

A new paradigm for phenotypic fingerprint construction was introduced, integrating phenotypic traits to generate interpretable phenotypic ID that enable the identification of different lettuce subpopulations. This provides a novel form of representation and theoretical foundation for phenotypic analysis, variety identification, and precise classification.

## 2 Materials and methods

### 2.1 Experimental materials

**Fig. 1:**
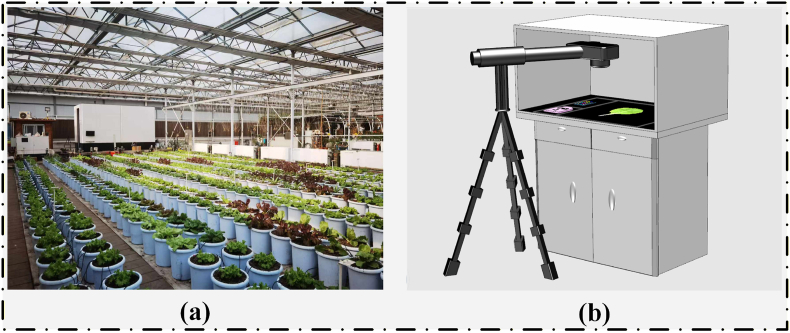
Experimental design and material acquisition. (a) Lettuce planting scene. (b) Image data acquisition platform.

A total of 237 lettuce varieties were cultivated in the greenhouse of the Beijing Academy of Agriculture and Forestry Sciences (BAAFS, 39.9438°N, 116.2876°E) between March 5 and May 11, 2023. Each variety was represented by three biological replicates. The plant materials covered seven subgroups: Butter (B), Crisphead (C), Leaf (L), Oakleaf (O), Romaine (R), Stem (S), and Wild relatives (W). All plants were grown under standardized greenhouse management to minimize environmental variation, including uniform irrigation, fertilization, and temperature regimes. At the mature stage, the most fully expanded and structurally representative leaf from each plant was selected for imaging, ensuring comparability of morphological features across subgroups. The lettuce planting scene is shown in Fig. 1a.

### 2.2 Image data acquisition and pre-processing

Image collection was carried out in a domestically manufactured standard light-source color assessment cabinet positioned adjacent to the greenhouse. The data acquisition platform is shown in Fig. 1b. A digital camera (77D, Canon Inc., Tokyo, Japan) was mounted on a stable tripod and equipped with a Canon EF-S 18–135 mm f/3.5–5.6 IS USM lens (focal length range 18–135 mm). A D65 international standard artificial daylight source (color temperature 6500 K, 18 W) was installed at the top of the cabinet to provide stable and uniform illumination, ensuring consistent lighting conditions across all samples and minimizing shadows and surface glare. All images were captured using fixed exposure parameters (ISO 100, shutter speed 1/125 s, aperture f/8) and a custom white-balance preset, with a resolution of 5328 × 4000 pixels. A Datacolor Spyder Checkr Photo Mini color calibration card was placed beside each leaf during imaging to facilitate color correction.

A total of 709 high-resolution leaf images were collected. The raw images were then processed through a standardized preprocessing workflow. First, to ensure accurate and comparable color-related trait measurements, color calibration was performed using Adobe Lightroom and the SpyderCheckr color profile. Although illumination conditions were strictly controlled, calibration was still required to correct for subtle chromatic deviations introduced by camera sensor characteristics, exposure metering adjustments, or long-term spectral drift. After color correction, each image was cropped to a fixed size of 2500 × 2500 pixels to remove redundant background and maintain consistent spatial dimensions across samples. The resulting images were used as the basis for multi-component semantic segmentation and subsequent phenotypic trait extraction of lettuce leaves.

### 2.3 Data annotation and enhancement

Pixel-level annotation was performed to generate ground-truth masks for the leaf multi-component semantic segmentation task. Manual annotation was conducted using LabelMe (v5.5.0), and three leaf components mesophyll, main vein, and lateral vein were delineated along with the background class. All labels were produced by trained annotators with experience in leaf morphology. Considering the fine-scale structural complexity of lateral veins, a subset of 102 representative images capturing the morphological diversity of the 237 varieties was selected for annotation. Consistency and standardization of the annotations were ensured through a unified annotation protocol that specified boundary definitions, delineation rules, and labeling criteria for all target components, thereby maintaining stable annotation quality across the dataset. To enhance model generalization and reduce overfitting, a comprehensive data augmentation strategy was applied to the annotated samples. The augmentation operations included rotation (±15°), horizontal and vertical flipping, scaling, translation, brightness jittering (±8%), and slight Gaussian blurring. After augmentation, the annotated dataset expanded to 1122 samples, providing a richer and more diverse dataset for model training.

**Fig. 2:**
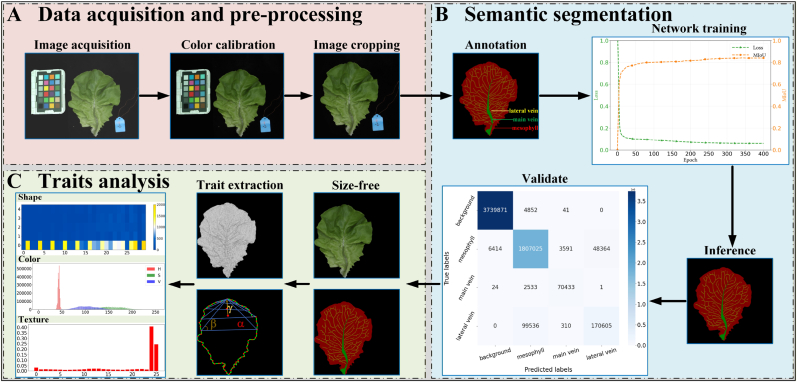
The flow chart of the phenotyping pipeline for lettuce leaves.

To this end, we developed a high-throughput phenotyping pipeline for lettuce leaves. This integrated workflow provides a robust framework for large-scale phenotypic analysis, enabling automated identification and quantitative evaluation of multiple semantic components. The overall processing steps, including image acquisition, preprocessing, annotation, semantic segmentation, and phenotypic trait extraction, are summarized in Fig. 2.

### 2.4 Semantic segmentation model

Accurate semantic segmentation of lettuce leaves faces unique challenges due to their complex morphological structure. The leaf contains broad mesophyll regions, dense hierarchical venation networks, and serrated or undulating margins. Lateral veins, in particular, form fine and irregular texture patterns that require precise boundary localization and robust multi-scale feature representation. The large variation in target size and the diversity of image content remain major bottlenecks for existing segmentation algorithms. Although many mainstream methods incorporate multi-branch architectures or multi-scale feature extraction, both strategies present limitations. Multi-branch designs may improve accuracy but significantly increase model parameters and computational cost, limiting their suitability for lightweight applications. Multi-scale schemes enhance cross-resolution feature aggregation, yet often fail to capture subtle micro-structural details such as delicate vein patterns, when relying on a single scaling strategy. These shortcomings can lead to incomplete venation delineation and blurred leaf margins, ultimately affecting the reliability of downstream phenotypic trait extraction.

**Fig. 3:**
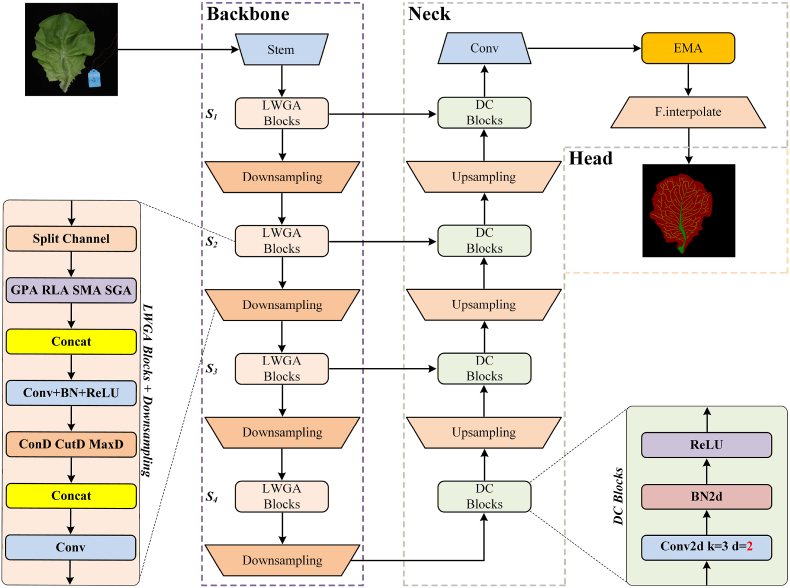
Architecture of LGASSNet model.

To address these challenges, we developed LGASSNet, a Lightweight Group Attention semantic segmentation network designed for multi-component lettuce leaf analysis. The network employs Lightweight Group Attention Network (LWGANet) [33] as its backbone to extract hierarchical feature representations. Shallow layers focus on local surface textures and fine venation details, enabling accurate delineation of lateral veins and serrated margins, whereas deeper layers capture the global morphological structure of the leaf. In the neck network, deep features are further refined through a Dilated Convolution (DC) blocks combined with skip connections. The DC blocks enlarges the receptive field while preserving spatial resolution, and the skip connections integrate features from all backbone stages, enhancing information flow and maintaining structural consistency across scales. Subsequently, the Efficient Multi-Scale Attention (EMA) [34] mechanism enhances contextual perception and strengthens the representation of intricate leaf structures. The fused multi-scale features are then restored to the original resolution through bilinear upsampling, after which the classification head generates pixel-level predictions for each leaf component. The overall architecture of LGASSNet is illustrated in Fig. 3.

#### 2.4.1 Backbone netork

The backbone of LGASSNet is built upon LWGANet [33], a lightweight architecture designed to balance multi-scale feature extraction with computational efficiency. It consists of four stages, where the spatial resolution is progressively reduced to 1/4, 1/8, 1/16, and 1/32 of the input size. Each stage contains an LWGA Blocks that divides the input feature X into four equal sub-feature maps {X_1_, X_2_, X_3_, X_4_}, which are processed by the GPA, RLA, SMA, and SGA attention modules [33], respectively. Through this grouped attention mechanism, the backbone can simultaneously capture fine-grained leaf textures, medium-scale venation structures, and long-range structural dependencies.

The GPA (gate point attention) [33] module is designed to process the input feature X_1_. Its primary goal is to capture fine-grained local features and enhance detail information in tiny structures. After channel transformation, BN, and ReLU activation, the feature representation is strengthened. A sigmoid activation function is then applied to generate the attention map, which is combined with the input through a residual connection to produce the output feature R_1_.

The RLA (regular local attention) [33] module processes the input feature X_2_. It focuses on extracting information efficiently from local regions. Standard convolution, followed by BN and ReLU activation, is applied to enhance the representation of local features. output feature R_2_.

The SMA (sparse medium-range attention) [33] module is applied to the input feature X_3_. It captures medium-range contextual information and is well-suited for extracting large and irregular features. The module first applies the TGFI [33] downsampling operation to obtain a reduced feature map. Based on this map, a sparse attention map is constructed and then upsampled through positional interpolation to restore the original spatial resolution. The final attention map is multiplied element-wise with X_3_ to produce the output feature R_3_.

The SGA (spare global attention) module [33] processes the input feature X_4_, aiming to capture long-range contextual information across the entire image. To maintain computational efficiency, the module adopts stage-specific processing strategies. In the first and second stages, where feature maps have large spatial dimensions, SGA employs a sparse global attention strategy. The TGFI module first downsamples X_4_ to obtain a reduced representation X_4_′. Standard convolution is then used to extract intermediate features, and dilated convolution is applied to generate a sparse global attention map. After element-wise multiplication, the result is upsampled using preserved coordinate information and combined with the original input through residual addition and BN normalization to form the output R_4_. In the third and fourth stages, the spatial resolution is much smaller, making full global processing unnecessary. Thus, SGA switches to a lightweight visual attention strategy, generating attention maps directly on the original feature map. These maps are multiplied element-wise with X_4_ and fused through residual connections to produce the final output feature R_4_.

To integrate the multi-scale information captured by the four attention branches, their output features are concatenated along the channel dimension to form the fused feature map. This fused representation then undergoes channel transformation, BN and ReLU to enhance feature expressiveness. Finally, a residual connection with the input X produces the output of the LWGA Blocks.

In the downsampling stages, the DRFD module [35] is introduced, which integrates three complementary strategies convolutional downsampling (ConD), cut-slice downsampling (CutD), and maxpooling downsampling (MaxD) [35]. This multi-path fusion preserves richer semantic and structural information while reducing computational complexity, thereby enhancing multi-scale feature learning and improving the extraction of key leaf components.

#### 2.4.2 Neck network

The neck network of LGASSNet refines the deep features generated by the backbone through a combination of DC Blocks and progressive stage-wise skip connections. The high-level features from stage 4 are first processed by a DC blocks, enabling the network to capture information from a larger receptive field while preserving spatial resolution. The resulting feature map is then upsampled and fused with the feature map from stage 3. This fused representation is further refined through another DC blocks, upsampled again, and sequentially merged with the feature maps from stage 2 and stage 1. Through this progressive fusion strategy, multi-scale information is continuously propagated and enhanced across hierarchical stages. Following the DC-based multi-stage refinement, an EMA [34] mechanism is applied. EMA uses parallel multi-scale convolutional paths and cross-space feature fusion to generate pixel-level attention maps that emphasize structurally informative regions such as lateral veins and serrated leaf margins. The resulting output of the neck network provides rich multi-scale feature representations for subsequent segmentation tasks.

#### 2.4.3 Loss function

In the multi-component segmentation of lettuce leaves, the vein regions are fine-grained and occupy only a small proportion of the image, which easily leads to class imbalance during training. To alleviate this issue, Focal Loss is employed to reduce the dominance of easily classified pixels and encourage the model to focus on hard-to-segment structures such as lateral veins. The Focal Loss is formulated as:

(1) $$L_{Focal}=-\alpha_{t}{\left(1-p_{t}\right)}^{\gamma }\;\log\left(p_{t}\right)$$

where $p_{t}$ denotes the predicted probability, $\alpha_{t}$ is the class-balancing factor, and γ is the focusing parameter (set γ=2 in this study).

To further preserve structural completeness and reduce boundary blurring, Dice Loss is introduced to emphasize region-level overlap between the predictions and ground truth:

(2) $$L_{Dice}=1-\frac{2\sum y_{true}y_{pred}}{\sum y_{true}+\sum y_{pred}}$$

where $y_{true}$ and $y_{pred}$ represent the ground truth and predicted values, respectively.

The final optimization objective is a weighted combination of the two losses:

(3) $$L=\lambda_{1}L_{Focal}+\lambda_{2}L_{Dice}$$

with $\lambda_{1}$ and $\lambda_{2}$ both set to 1 in this study. This joint loss encourages balanced learning across components while improving the segmentation accuracy of fine venation structures.

### 2.5 Phenotypic traits analysis

Based on the semantic segmentation results, phenotypic traits of lettuce leaves were automatically extracted from multiple dimensions, including shape, color, and texture. Lettuce leaves contain both size-related and size-free traits. To eliminate the influence of leaf size variation and improve the consistency of phenotypic analysis, we adopted the normalization approach proposed by Du et al. [23]. This method standardizes leaf geometry through rotation and scaling, enabling the generation of comparable “size-free” representations across different varieties. Following normalization, each leaf image was represented at a resolution of 1080 × 1080 pixels to balance analytical precision and computational efficiency. The extracted phenotypic traits and their definitions are summarized in Table S1, which encompasses leaf shape, color characteristics, and texture features.

#### 2.5.1 Shape traits analysis

##### 2.5.1.1 Multiscale triangle descriptor

The multiscale triangle descriptor (MTD) is a shape descriptor based on leaf contours. It is designed to capture geometric characteristics of leaves, such as curvature, symmetry, and local shape variation, while ensuring invariance to translation, rotation, and scale [36]. First, multiscale triangles are constructed based on the segmented leaf contours. The outermost contour is extracted and uniformly sampled into N points, resulting in a point sequence $S=\left\{P_{i}\left(x_{i},y_{i}\right),i=1,2,\ldots ,N\right\}$. The number of scales is defined as $T_{s}=\lfloor \log_{2}\left(N/2\right)\rfloor$. At each scale k, the sampling interval is $d\left(k\right)=2^{k-1}$. For each point $P_{i}$, a triangle is formed with its two neighboring points $P_{i-d\left(k\right)}$ and $P_{i+d\left(k\right)}$. The signed triangle area (TSA) at scale k is calculated as:

(4) $${TSA}_{k}\left(i\right)=\frac{1}{2}\left|\begin{array}{ccc} \begin{array}{c} x_{i-d\left(k\right)}\\ \begin{array}{c} x_{i}\\ x_{i+d\left(k\right)} \end{array} \end{array} & \begin{array}{c} y_{i-d\left(k\right)}\\ \begin{array}{c} y_{i}\\ y_{i+d\left(k\right)} \end{array} \end{array} & \begin{array}{c} 1\\ \begin{array}{c} 1\\ 1 \end{array} \end{array} \end{array}\right|$$

The degree of bend at point $P_{i}$ is defined as $\alpha_{k}\left(i\right)=|{TSA}_{k}\left(i\right)|$ and the convex or concave is represented by $\beta_{k}\left(i\right)=sgn\left({TSA}_{k}\left(i\right)\right)$.

Next, the center distance of the triangle is computed as:

(5) $$\gamma_{k}\left(i\right)=\sqrt{{\left(x_{i}-x_{gik}\right)}^{2}+{\left(y_{i}-y_{gik}\right)}^{2}}$$

where $\left(x_{gik},y_{gik}\right)$ is the centroid of the triangle:

(6) $$\left\{ \begin{array}{c} x_{gik}=\left(x_{i-d\left(k\right)}+x_{i}+x_{i+d\left(k\right)}\right)/3\\ y_{gik}=\left(y_{i-d\left(k\right)}+y_{i}+y_{i+d\left(k\right)}\right)/3 \end{array}\right.$$

Each point is then described by a triangle feature vector $\text{MTD}\left(P_{i}\right)=\left\{\alpha \left(i\right),\beta \left(i\right),\gamma \left(i\right),i=1,2,\ldots ,N\right\}$. The full multiscale triangle descriptor for the leaf contour is:

(7) $$MTD\left(S\right)={\left(MTD\left(P_{1}\right),\ldots ,MTD\left(P_{N}\right)\right)}^{T}\;\;\;\;\;\;\;\;\;\;\;\;\;\;\;=\left(\begin{array}{ccc} \alpha \left(P_{1}\right) & \beta \left(P_{1}\right) & \gamma \left(P_{1}\right)\\ \vdots & \ddots & \vdots \\ \alpha \left(P_{N}\right) & \beta \left(P_{N}\right) & \gamma \left(P_{N}\right) \end{array}\right)\;\;=\left(\alpha ,\beta ,\gamma \right)$$

To ensure scale invariance, the local normalization of $\alpha_{k}\left(i\right)$ and $\gamma_{k}\left(i\right)$ [37]:

(8) $$\alpha_{k}\left(i\right)=\frac{\alpha_{k}\left(i\right)}{\max_{i=1}^{N}\left|\alpha_{k}\left(i\right)\right|}$$

(9) $$\gamma_{k}\left(i\right)=\frac{\gamma_{k}\left(i\right)}{\max_{i=1}^{N}\left|\gamma_{k}\left(i\right)\right|}$$

To eliminate variations due to different starting points, a discrete Fourier transform (DFT) is applied to the sequences of α,β,γ at each scale. Only the first M low-frequency components are retained to reduce noise and enhance rotation invariance. The final MTD is defined as:

(10) $$\text{MTD}\left(S\right)=\left\{\left(\alpha_{t}\left(v\right),\beta_{t}\left(v\right),\gamma_{t}\left(v\right)\right)|t=1,\ldots ,T_{s},v=1,\ldots ,M\right\}\;\;=\left(\alpha^{'},\beta^{'},\gamma^{'}\right)$$

##### 2.5.1.2 Leaf shape estimation

Leaf shape is a critical trait that directly reflects photosynthetic efficiency and water management capabilities. However, the diverse morphologies and varying sizes of lettuce leaves present challenges for quantitative extraction. To address this, we propose a radial distance-based method that assumes an ideal lettuce leaf shape to be circular, where the Euclidean distances from the leaf center to its boundary points are approximately equal. Specifically, the leaf is divided into n equal segments (n=8), and the euclidean distances from the leaf center to each segmentation point are computed. These distances are then used to quantify the degree of similarity between the actual leaf shape and the ideal circular form. The detailed steps of this method are as follows:

First, we perform a circular check. Based on the concept of leaf-class, we calculate the ratio of leaf length to leaf width, denoted as LWr=LL/LW. If LWr∈(0,1.2], the center point O of the leaf is located, and the radial distance $d_{1},d_{2},..,d_{n}$ are calculated by measuring the distance from O to various segmentation points $P_{i}$ along the leaf's edge. The resulting radial distances are as follows:

(11) $$d_{i}=\sqrt{{\left(x_{i}-x_{0}\right)}^{2}+{\left(y_{i}-y_{0}\right)}^{2}},i=1,2,\ldots ,n$$

where $\left(x_{0},y_{0}\right)$ denotes the coordinates of the leaf center O. $\left(x_{i},y_{i}\right)$ represents the coordinates of the ith segmentation point $P_{i}$, and $d_{i}$ denotes the Euclidean distance from the leaf center to the i−th segmentation point. The standard deviation of the radial distance $\sigma_{d}$, is then calculated to quantify the leaf's approximation to a circular shape, as given by the following equation:

(12) $$\sigma_{d}=\sqrt{\frac{1}{n}\sum_{i=1}^{n}{\left(d_{i}-\bar{d}\right)}^{2}}$$

(13) $$\bar{d}=\frac{1}{n}\sum_{i=1}^{n}d_{i}$$

where $\bar{d}$ represents the mean value of the radial distances. The closer the value of $\sigma_{d}$ is to 1, the more circular the shape of the leaf. Based on the thresholds, leaves are classified as follows: narrow oblate $\sigma_{d}\in (0,0.8]$, circular $\sigma_{d}\in [0.8,1.5)$ and medium oblate $\sigma_{d}\in [1.5,+\infty )$.

Next, the elliptic shape is assessed. If LWr∈(1.2,3.0], the classification is based on the following thresholds: broad elliptic LWr∈(1.2,1.8], medium elliptic LWr∈(1.8,2.4], narrow elliptic LWr∈(2.4,3.0].

Thirdly, the linear and lanceolate are assessed. If LWr∈(3.0,+∞), the leaf apex and the 50th points on both the left and right sides are selected to form the pinch angle. The pinch angle θ is then calculated. Based on the thresholds, lanceolate is classified for θ∈(0°,90°], and linear for θ∈(90°,180°].

Additionally, at each step, broad obtrullate and triangular classifications were made based on the side relationship theorem. Finally, leaf shapes were categorized as narrow oblate, circular, medium oblate, broad elliptic, medium elliptic, narrow elliptic, linear, lanceolate, broad obtrullate and triangular, following the UPOV guidelines.

##### 2.5.1.3 Leaf tip shape estimation

The leaf tip shape primarily refers to the morphological characteristics of the leaf's apex, which reflect the plant's growing environment and physiological state. It is also a crucial trait for plant classification. To analyze the leaf tip shape, the top 1/5 portion of the leaf is extracted as the region of interest. The concave and convex contour angles are then calculated by approximating the polygonal contour. Let $P_{c}\left(x_{c},y_{c}\right)$ denote the coordinates of the tip of the leaf, $P_{l}\left(x_{l},y_{l}\right)$ represent the coordinates of the 50th point adjacent to the left, and $P_{r}\left(x_{r},y_{r}\right)$ denote the coordinates of the 50th point adjacent to the right. The pinch angle θ is computed using the following equation:

(14) $$\begin{array}{c} \theta =\alpha \;\tan\;2(\left(x_{l}-x_{c}\right)\left(y_{r}-y_{c}\right)-\left(y_{l}-y_{c}\right)\left(x_{r}-x_{c}\right)\text{,}\\ \left(x_{l}-x_{c}\right)\left(x_{r}-x_{c}\right)+\left(y_{l}-y_{c}\right)\left(y_{r}-y_{c}\right))\cdot \frac{180}{\pi } \end{array}$$

where θ∈[−180°,180°]. To more intuitively represent the degree of concavity and convexity of the leaf, the angle is converted to the range [0°,360°] using the following formula:

(15) $$\theta_{adjusted}=\left(\theta +360\right)\;\mathrm{mod}\;360$$

Leaf tip shapes are classified into acute $(\theta_{adjusted}\in \left(0{}^{\circ},120{}^{\circ}]\right)$, obtuse $(\theta_{adjusted}\in \left(120{}^{\circ},150{}^{\circ}]\right)$, rounded $(\theta_{adjusted}\in \left(150{}^{\circ},200{}^{\circ}]\right)$, and obcordate $(\theta_{adjusted}\in \left(200{}^{\circ},360{}^{\circ}]\right)$ based on angular differences.

##### 2.5.1.4 Leaf margin shape estimation

Leaf margins reflect a plant's ability to adapt to wind, light, or arid environments. First, we extracted the edge contour coordinate points and applied an interval sampling method to calculate the angles between neighboring points (i.e., the 10th coordinate point on the left and right) cyclically, as described in Eqs. (14), (15). Using the flat angle as the critical for division, if the angle is less than 180°, the margin is concave, with greater distances indicating a more intense concavity. Conversely, if the angle exceeds 180°, the margin is convex, with larger distances signifying more pronounced convexity. Based on the degree of intensity (with 15° as the classification threshold in the experiment) and the proportion of the leaf margins, the leaf margins were classified as crenate, dentate, bidentate, and tridentate.

##### 2.5.1.5 Leaf veins shape estimation

Leaf vein distribution reflects the physiological health of lettuce. It not only supports plant growth and nutrient transport but also provides valuable insights into nutritional status and environmental adaptation. The complex structure of leaf veins in mature plants presents challenges for accurate extraction. Moreover, determining the angles between leaf veins is a significant difficulty. The Hough line Transform is a widely used method for detecting straight lines in images. It is particularly effective for detecting leaf vein lines and can efficiently extract and analyze the angular information of these veins. The principle of angle extraction is based on the polar coordinate representation of a straight line. In a Cartesian coordinate system, the equation of a straight line is given by y=kx+b, where k represents the slope and b is the y-intercept. To apply the Hough Transform, we convert this equation to its polar form: ρ=xcosθ+ysinθ, where ρ is the distance from the origin to the line, and θ is the angle of the line's normal. For each point (x,y) in the image, we can compute all possible combinations of ρ and θ based on the above polar equation. This allows each point in the image to be mapped to a series of linear parameters in polar coordinate space.

The steps are as follows: First, for each edge point (x,y), different values of θ are selected to compute the corresponding ρ=xcosθ+ysinθ. Then, the number of votes for each pair (ρ,θ) in the parameter space (ρ,θ) is recorded. The angle θ corresponding to the maximum vote is used to determine the angular α-transformation of a straight line, as shown in Eq. (16). Finally, by performing mean normalization, the leaf vein shapes are classified into the following categories based on threshold values: flabellate $(\alpha_{std}\in (0{}^{\circ},100{}^{\circ}]\&\alpha_{mean}\in \left(0{}^{\circ},90{}^{\circ}]\right)$, semi_flabellate $(\alpha_{std}\in (0{}^{\circ},100{}^{\circ}]\&\alpha_{mean}\in \left(90{}^{\circ},100{}^{\circ}]\right)$, and not_flabellate (others). Here, $\alpha_{std}$ represents the leaf vein shape standard deviation (LVSs), and $\alpha_{mean}$ represents the leaf vein shape mean (LVSm).

(16) $$\alpha =\theta \times \frac{180}{\pi }$$

#### 2.5.2 Color traits analysis

Leaf color directly reflects key physiological traits such as chlorophyll content, anthocyanin content, photosynthetic efficiency, and environmental adaptability. To more intuitively capture the hue and brightness of the leaf, the RGB image was converted to the HSV color space, where the H, S, and V components were averaged. Based on predefined threshold values, leaf color was categorized into yellowish green, green, and greyish green. Color brightness was classified into five levels: very dark, dark, medium, light, and very light. Additionally, the degree of anthocyanin pigmentation was assessed.

#### 2.5.3 Texture traits analysis

LBP-HF is a rotation-invariant texture descriptor designed to capture microstructural features of leaf surfaces, including veins, spots, and surface roughness [38]. The method begins with the extraction of standard Local Binary Patterns (LBP) features. For each central pixel $P_{0}\left(x,y\right)$, P neighboring pixels are sampled on a circular path with radius R, denoted as ${\left\{P_{p}\right\}}_{p=0}^{P-1}$. The LBP code for each pixel is calculated as follows:

(17) $${LBP}_{P,R}\left(x,y\right)=\sum_{p=0}^{P-1}s\left(f\left(x,y\right)-f\left(x_{p},y_{p}\right)\right)*2^{P}$$

where s(u) is the thresholding function:

(18) $$s\left(u\right)=\left\{ \begin{array}{c} 1\;if\;u\geq 0\\ 0\;if\;u<0 \end{array}\right.$$

When the LBP pattern exhibits at most two transitions between 0 and 1, it is classified as a uniform pattern. A histogram is then constructed by counting the occurrences of these uniform patterns, denoted as $h_{I}\left(U_{P}\left(n,r\right)\right)$, where n indexes the type of pattern and r refers to the radial location. To enhance rotation invariance, a DFT is applied along the histogram rows:

(19) $$H\left(n,u\right)=\sum_{r=0}^{P-1}h_{I}\left(U_{P}\left(n,r\right)\right)e^{-i2\pi ur/P}$$

The rotation-invariant LBP feature is then obtained by extracting the magnitude of the DFT coefficients |H(n,0)|. The final LBP-HF descriptor includes both the DFT-based frequency features and the original LBP histogram values. The complete feature vector is expressed as:

(20) $$\begin{array}{c} {fv}_{LBP-HF}=\lfloor \left|H\left(1,0\right)\right|,\ldots ,\left|H\left(1,P/2\right)\right|\text{,}\\ \ldots \text{,}\\ \left|H\left(P-1,0\right)\right|,\ldots ,\left|H\left(P-1,P/2\right)\right|\text{,}\\ h\left(U_{p}\left(0,0\right)\right),h\left(U_{p}\left(P,0\right)\right),h\left(U_{p}\left(P+1,0\right)\right)\rfloor \end{array}$$

### 2.6 Phenotypic fingerprint construction

**Fig. 4:**
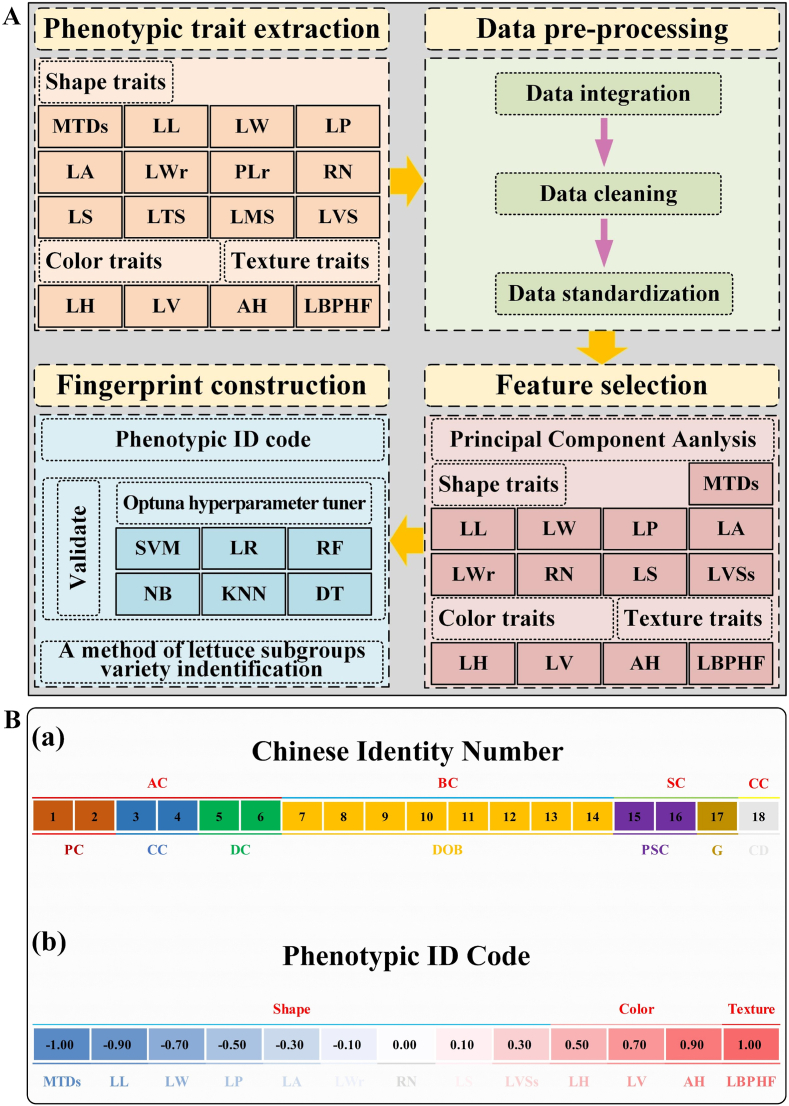
Phenotypic fingerprint construction. (A) The flow chart of the phenotypic fingerprint construction. (B) Phenotypic ID code based on chinese identity number.

In recent years, phenotypic fingerprinting has emerged as a powerful complement to molecular marker technologies, demonstrating broad potential in crop variety identification, particularly in scenarios where molecular detection is unavailable [[39], [40], [41]]. We propose a phenotypic fingerprinting framework based on leaf morphological traits, aiming to develop an efficient, robust, and highly discriminative method for lettuce variety identification. As illustrated in Fig. 4A, the method consists of four key steps: phenotypic data integration, data standardization, feature selection, and fingerprint construction and validation. First, phenotypic traits from multiple semantic components were integrated to establish a multidimensional comprehensive trait profile. The dataset was then standardized by calculating the mean and standard deviation of each trait, and outliers were removed using an improved Z-score method (with |Z|>3 as the threshold), enhancing data quality and robustness. To eliminate the influence of dimensional inconsistencies among traits, Min-Max normalization was applied, linearly mapping all values to the range [−1,1], thereby ensuring comparability across traits. PCA (Principal component analysis) was then employed to reduce data dimensionality and select representative traits. Based on the ranking of principal component eigenvalues and scree plot analysis, core traits that preserve the most phenotypic information while maximizing variety differentiation were selected to construct phenotypic fingerprints for each variety. Furthermore, we introduce a novel visualization approach for encoding these phenotypic ID, as shown in Fig. 4B. This method converts the normalized trait into unique vectors or heatmap coding sequence, preserving the biological significance of each trait while enhancing interpretability and usability in variety identification.

### 2.7 Subgroup variety identification

Phenotypic ID features were used as input variables to develop classification models for subpopulation variety identification. Six machine learning algorithms were employed: Decision Tree (DT), K-Nearest Neighbors (KNN), Naive Bayes (NB), Random Forest (RF), Logistic Regression (LR), and Support Vector Machine (SVM). However, uncertainty arising from model structure and parameter settings may cause the predictions to poorly reflect the actual relationships between variables. To improve model automation and classification accuracy, we adopted Optuna [42], a Bayesian optimization framework for hyperparameter tuning. Optuna optimizes model performance by maximizing validation accuracy and F1-score during training and evaluation. Compared to traditional manual tuning, this method increases optimization efficiency, reduces variability caused by subjective parameter choices, and enhances model generalization.

### 2.8 Evaluation metrics

In this study, multiple evaluation metrics were adopted according to the characteristics of different tasks. For semantic segmentation, DUS test trait verification, and subgroup classification, all of which are category discrimination tasks. Accuracy, Precision, Recall, and F1-score were used as the primary metrics. Their definitions are:

(21) $$\text{Accuracy}\left(A\right)=\frac{TP+TN}{TP+FP+TN+FN}$$

(22) $$\text{Precision}\left(P\right)=\frac{TP}{TP+FP}\times 100\%$$

(23) $$\text{Recall}\left(R\right)=\frac{TP}{TP+FN}\times 100\%$$

(24) $$F1-\text{score}\left(F1\right)=\frac{2\mathrm{PR}}{P+R}\times 100\%$$

where TP (True Positive) refers to the number of positive samples correctly predicted as positive, TN (True Negative) refers to the number of negative samples correctly predicted as negative, FP (False Positive) refers to the number of negative samples incorrectly predicted as positive, and FN (False Negative) refers to the number of positive samples incorrectly predicted as negative. To further assess spatial overlap and structural consistency in semantic segmentation, the Mean Intersection over Union (MIoU) metric was introduced.

(25) $$\text{MIoU}=\frac{1}{N}\sum_{i=0}^{N}\frac{\text{TP}}{\text{TP}+\text{FP}+\text{FN}}\times 100\%$$

For geometric trait validation, extraction accuracy was evaluated using the coefficient of determination (R^2^), Root Mean Square Error (RMSE), and Mean Absolute Percentage Error (MAPE):

(26) $$R^{2}=1-\frac{\sum {\left(y_{i}-{\hat{y}}_{i}\right)}^{2}}{\sum {\left(y_{i}-\bar{y}\right)}^{2}}$$

(27) $$\text{RMSE}=\sqrt{\frac{1}{n}\sum {\left(y_{i}-{\hat{y}}_{i}\right)}^{2}}$$

(28) $$\text{MAPE}=\frac{100}{n}\sum \left|\frac{y_{i}-{\hat{y}}_{i}}{y_{i}}\right|$$

where $y_{i}$ represents manual measurements and ${\hat{y}}_{i}$ denotes predicted values. R^2^ measures goodness of fit, while RMSE and MAPE quantify the magnitude of extraction errors.

### 2.9 Experimental setup

The training platform used in this study was a Windows 11 (64-bit) operating system. Hardware parameter settings:CPU Intel(R) Core(TM) i7-12700 @ 2.10 GHz operating memory, 32 GB of RAM, 1 TB of SSD, and 8 GB of NVIDIA GeForce RTX 3070 Ti GPU. Anaconda 3.5.0 (Anaconda Inc., USA), CUDA 11.1 (Nvidia, USA), and cuDNN 8.0.4 (Nvidia, USA) libraries were used. In addition, the open-source deep learning framework Pytorch was used as the development environment, and the programming language used was Python 3.8.5 (Python Software Foundation, USA). The software used to train the model was PyCharm 2022.01. To ensure reliable model evaluation, the dataset was randomly divided into 80% training and 20% validation sets using a fixed random seed to guarantee reproducibility. In addition, a 5-fold cross-validation strategy was employed during hyperparameter tuning to assess model stability under different data partitions. The final model was trained using the optimal hyperparameters obtained from cross-validation, as summarized in Table S2.

## 3 Results

### 3.1 Semantic segmentation performance analysis

To comprehensively assess the effectiveness of LGASSNet in multi-component lettuce leaf segmentation, we performed a series of quantitative and qualitative experiments. The evaluation included comparisons with mainstream semantic segmentation models, analysis of lightweight efficiency, examination of convergence behavior, and visualization of multi-component segmentation results.

As shown in Table 1, LGASSNet achieves the best overall segmentation performance among all evaluated models, with an Accuracy of 96.78%, Precision of 91.24%, Recall of 89.13%, and an F1-score of 90.08%. Compared with CNN-based architectures such as PSPNet, DeepLabv3+, HRNet, and UNet, LGASSNet provides notable improvements across both pixel-level and structure-aware metrics. In particular, it surpasses UNet and HRNet by 3.71% and 5.18% in F1-score, respectively, reflecting stronger adaptability to complex leaf textures and intricate edge structures. Variants adopting different backbone designs, including VGG-DC-EMA and Resnet-DC-EMA, also perform competitively. However, LGASSNet achieves an additional 0.71–2.45% increase in Recall, indicating enhanced capability in covering fine structural regions and maintaining boundary continuity.

**Table 1:** Performance comparisons of different backbone and other models.

Model	Accuracy↑	Precision↑	Recall↑	F1-score↑
PSPNet	94.35	70.65	73.47	72.19
DeepLabv3+	95.53	87.82	81.84	83.64
HRNet	95.66	87.81	83.32	84.90
UNet	95.88	87.84	85.29	86.37
VGG-DC-EMA	96.14	88.36	87.10	87.63
Resnet-DC-EMA	96.46	89.79	88.42	89.03
LGASSNet(ours)	**96.78**	**91.24**	**89.13**	**90.08**

As shown in Table 2, LGASSNet exhibits excellent lightweight efficiency, containing only 4.58M parameters with a computational cost of 22.27G FLOPs. While maintaining an inference speed of 63.35 FPS, it still achieves the highest MIoU of 84.08%, demonstrating a well-balanced trade-off between accuracy and computational cost. Although Resnet-DC-EMA attains the highest frame rate of 64.46 FPS, its configuration requires 51.06M parameters and 51.25G FLOPs, resulting in substantially increased model complexity. In contrast, LGASSNet provides a more reliable balance for phenotyping applications, maintaining fine-grained segmentation quality while supporting real-time processing. Compared with models such as DeepLabv3+ (54.71M parameters, 167.24G FLOPs) and UNet (450.89G FLOPs), LGASSNet reduces computational cost by approximately 87% to 95% and lowers parameter count by 82% to 92%. This reduction underscores its suitability for deployment in resource-limited or embedded phenotyping systems.

**Table 2:** Lightweight performance comparisons of different models.

Model	MIoU↑	Parameters(M)↓	FLOPs(G)↓	FPS↑
PSPNet	68.21	49.07	123.46	63.83
DeepLabv3+	76.46	54.71	167.24	47.38
HRNet	77.64	29.54	91.35	29.76
UNet	79.02	24.89	450.89	26.72
VGG-DC-EMA	80.55	30.28	190.70	32.40
Resnet-DC-EMA	82.58	51.06	51.25	**64.46**
LGASSNet(ours)	**84.08**	**4.58**	**22.27**	63.35

**Fig. 5:**
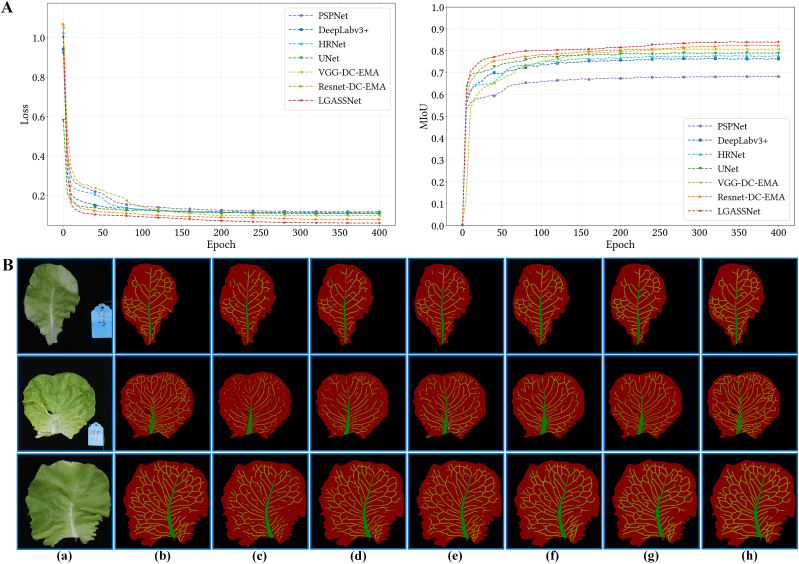
Performance comparison of segmentation model. (A) Training loss and MIoU curves. (B) Qualitative segmentation results. (a) input image, (b) ground truth, (c) DeepLabv3+, (d) HRNet, (e) UNet, (f) VGG-DC-EMA, (g) Resnet-DC-EMA, (h) LGASSNet (ours).

Fig. 5A illustrates the training loss and MIoU curves. LGASSNet exhibits fast and stable convergence, with the training loss decreasing smoothly and eventually reaching a steady plateau without oscillation. The MIoU curve increases consistently throughout training and stabilizes at a value higher than those of the other models, demonstrating the strong generalization capability of the network. This rapid convergence benefits from the synergy among LWGANet's group attention mechanism, the receptive-field expansion provided by the DC blocks, and the cross-scale feature refinement introduced by the EMA module, which together enable effective optimization of fine structural regions.

Fig. 5B illustrates the qualitative segmentation results. Although most models correctly capture the overall leaf contour, noticeable differences emerge in structurally complex regions such as serrated margins and lateral veins. CNN-based architectures (e.g., DeepLabv3+, HRNet, and UNet) often exhibit incomplete vein segmentation or discontinuous boundaries. In contrast, models incorporating both DC and EMA modules (VGG-DC-EMA and Resnet-DC-EMA) show marked improvements in edge continuity and structural consistency, reflecting stronger sensitivity to fine-scale details. LGASSNet delivers the most stable performance across all components, including mesophyll, main veins, and lateral veins. It accurately delineates primary and secondary venation while preserving margin features, achieving close alignment with manual annotations. This visual advantage supports downstream phenotypic trait extraction, particularly for traits dependent on venation topology and leaf-margin morphology.

Overall, the results demonstrate that LGASSNet achieves top-level performance in accuracy, structural completeness, computational efficiency, and real-time capability, highlighting its strong potential for large-scale automated lettuce phenotyping.

### 3.2 Analysis results of phenotypic traits

**Fig. 6:**
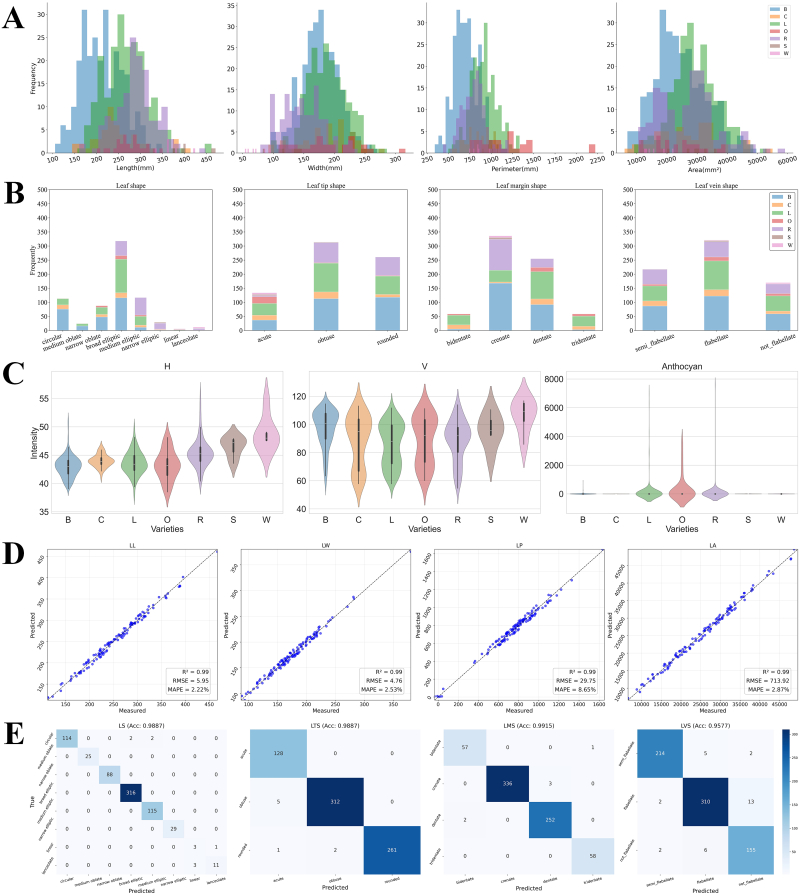
Analysis results of phenotypic traits. (A) Analysis results of basic phenotypic traits. (B) Analysis results of DUS test traits about morphological. (C) Analysis results of DUS test traits about color. (D) Validation of geometric measurements. (E) Validation of DUS test trait classification.

The basic phenotypic traits of 709 lettuce leaves were analyzed in Fig. 6A. The phenotypic characteristics varied significantly among different varieties. Leaf length ranged from 105.4 mm to 470.32 mm, with an average leaf length of 251.81 mm. The shortest one was the Butter B-165-3, collected in 1963 from Warszawa Poland. The longest one was the Stem S-1193-2, collected in 2009 from China. Leaf width ranged from 53.73 mm to 316.17 mm, with an average leaf width of 173.52 mm. The narrowest one was the Wild relatives W-1264-2, collected in 1961 from England United Kingdom. The widest one was the Oakleaf O-901-2, collected in 1989 from United States. Leaf perimeter ranged from 337.91 mm to 2222.09 mm, with an average leaf perimeter of 817.67 mm. The shortest one was the Butter B-165-3, collected in 1963 from Warszawa Poland. The longest one was the Oakleaf O-901-2, collected in 1989 from United States. Leaf area ranged from 5961.42 mm^2^ to 59172.82 mm^2^, with an average leaf area of 26449.94 mm^2^. The smallest leaf area was the Crisphead C-384-2, collected in 1989 from California United States. The largest leaf area was the Roman R-1054-1, collected in 1948, from India. The leaf length, leaf width, leaf perimeter and leaf area were all normally distributed.

The results of the 709 lettuce leaf shape (circular, medium oblate, narrow oblate, broad elliptic, medium elliptic, narrow elliptic, linear, lanceolate), leaf tip shape (acute, obtuse, rounded), leaf margin shape (bidentate, crenate, dentate, tridentate), and leaf vein shape (semi_flabellate, flabellate, not_flabellate) DUS test trait analysis (In this study, only the morphology present in the experimental material is analyzed). as shown in Fig. 6B. In terms of leaf shape, B varieties were mainly circular, medium oblate, narrow oblate, broad elliptic, and medium elliptic. R varieties were primarily broad elliptic, medium elliptic, and narrow elliptic. S varieties mostly exhibited linear shapes, while W varieties tended to be lanceolate. For leaf tip shape, B, L, and R varieties were dominated by obtuse and rounded tips. C varieties mainly showed acute and obtuse tips, whereas other varieties were predominantly acute. Regarding leaf margin shape, B and R varieties were primarily crenate and dentate. L varieties mostly exhibited dentate margins. C varieties frequently showed bidentate margins. Other varieties were mostly crenate. In terms of leaf vein shape, the majority of varieties exhibited a flabellate pattern.

The DUS test traits of hue, brightness, and anthocyanin content were analyzed for 709 lettuce varieties, as shown in Fig. 6C. The overall hue (H) ranged from 40° to 50°, and the brightness (V) ranged from 80° to 120°, indicating a predominant yellowish green hue and medium brightness. Among the varieties, Butter exhibited the lowest overall hue (H) value, displaying a yellowish green color, while Wild Relatives had the highest hue (H) value, resulting in a green color. In terms of brightness, Leaf displayed the lowest overall brightness (V) value, appearing dark, while Wild Relatives had the highest brightness (V) value, showing medium brightness. Regarding anthocyanin content, Leaf, Oakleaf, and Roman varieties exhibited clear anthocyanin distributions, while the remaining varieties contained either trace amounts or no detectable anthocyanin.

To validate the reliability of the extracted phenotypic traits, we performed both geometric and DUS test trait verification. For geometric traits (LL, LW, LP, LA), manual measurements of 120 leaves were obtained using ImageJ and compared with the pipeline-derived values, as illustrated in Fig. 6D. The results showed strong consistency (R^2^ > 0.99, MAPE < 8.65%), confirming the accuracy of quantitative extraction. For DUS test traits (LS, LTS, LMS, LVS), annotations from two domain experts were used as reference labels, and the corresponding confusion matrix is shown in Fig. 6E. The evaluation achieved an overall accuracy of 95.77%, with F1-scores above 0.96 for all classes, indicating that the proposed pipeline reliably captures morphological categories. These results collectively demonstrate the robustness and biological validity of both geometric and DUS trait extraction.

To quantitatively verify whether the extracted phenotypic traits differed significantly among lettuce subgroups, we performed a comprehensive one-way ANOVA followed by Tukey's HSD post-hoc test across all 17 traits, as shown in Table S3. The standardized mean values of each trait for the seven subgroups are visualized in Fig. S1A. The heatmap reveals clear subgroup-specific phenotypic patterns, particularly in morphological traits such as LTS, LVSs, LVSm, LMS, LL, LW, and LA. ANOVA results indicated that 16 of the 17 traits exhibited significant differences among subgroups (p < 0.05), including 11 traits with highly significant variation (p < 0.001). These results confirm that phenotypic divergence across subgroups is statistically meaningful rather than arising from sampling variation. To further evaluate which subgroup pairs differed significantly, we performed Tukey's HSD multiple-comparison test, which revealed extensive pairwise separation patterns. Subgroups such as R and C exhibited consistent and highly significant differences across multiple traits, forming well-defined phenotypic clusters. In contrast, the W subgroup showed the greatest within-group variation and broader overlap with other groups, reflecting its higher level of genetic heterogeneity. The Tukey significance matrix in Fig. S1B demonstrates that most subgroup pairs differ significantly across a wide range of traits, indicating that the phenotypic divergence described in this study is strongly supported by statistical evidence. Together, the ANOVA and Tukey HSD analyses validate the robustness of phenotype-based differentiation among lettuce subgroups.

After confirming that phenotypic traits exhibited significant subgroup-level divergence, we further examined the internal relationships among the 17 traits through Pearson correlation analysis. As shown in Fig. S1C. Significant positive and negative correlations were observed among different traits. MTDs showed a significant positive correlation with LS, indicating that local structural variations directly influence the development of overall leaf morphology. LBPHF exhibited strong positive correlations with several geometric traits, including LL, LW, LP, and LA. Notably, its correlation with LA reached as high as *r* = 0.99. This suggests that texture complexity increases with leaf size, indicating a close association between leaf surface texture and linear dimensions. Conversely, LBPHF showed a significant negative correlation with LV, implying that increased structural complexity may hinder the even distribution of pigments, resulting in lower brightness. This indicates a potential antagonistic relationship between texture richness and pigment accumulation. Strong positive correlations were also observed among fundamental geometric traits such as LL, LW, LP, and LA. For instance, LL and LA showed a correlation of *r* = 0.64, and LW and LA showed *r* = 0.71. These results reflect coordinated expansion among linear dimensions during leaf growth.Among ratio-based traits. LWr was negatively correlated with LW (*r* = −0.63), indicating that as leaf width increases, the length-to-width ratio decreases. This trend suggests a morphological shift towards a rounder leaf shape. PLr exhibited a significant negative correlation with RN (*r* = −0.60), suggesting that rounder leaves tend to have a more compact contour, with a lower perimeter-to-diameter ratio. Moderate positive correlations were identified among structural traits such as LS, LTS, and LMS. These relationships imply a coordinated change across the overall leaf shape, leaf tip shape, and leaf margin shape. A significant negative correlation was also found between LV and AH (*r* = −0.58), indicating that leaves with lower brightness typically contain higher levels of anthocyanins. This is consistent with the known light-absorbing and color-deepening properties of anthocyanins. The diagonal density plots illustrated the distribution of each phenotypic trait. Most traits followed an approximately normal distribution, supporting the reliability and consistency of the data. In summary, the correlation analysis reveals multiple synergistic patterns among leaf traits. Interactions between shape, color, texture, and pigment accumulation suggest complex regulatory relationships. These relationships were distinguished by constructing phenotypic fingerprints.

### 3.3 Construction and visualization of phenotypic fingerprint

**Fig. 7:**
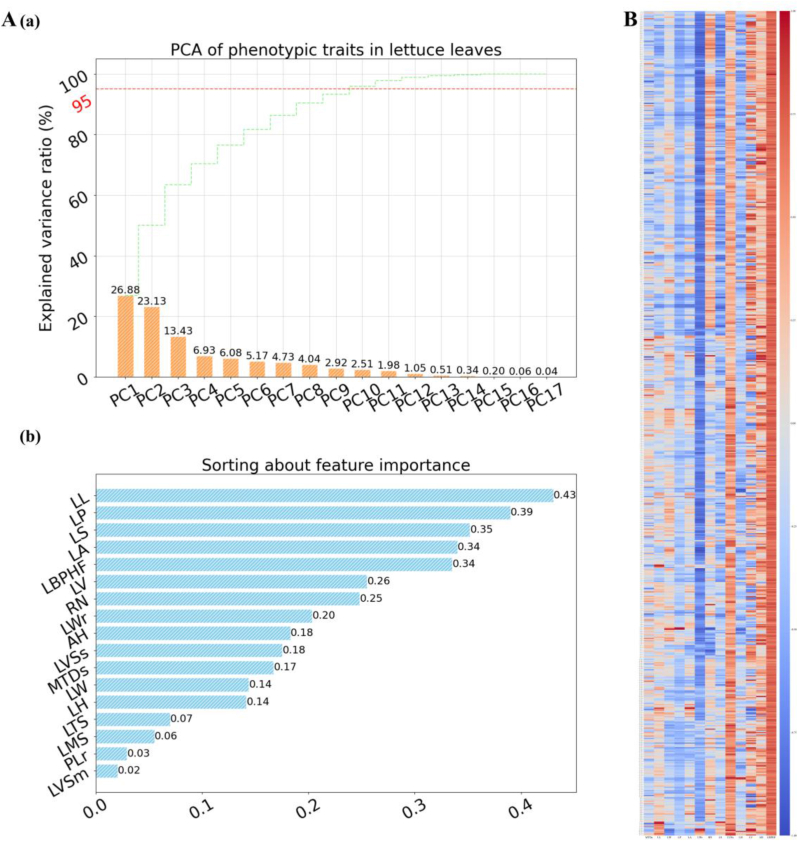
Construction and visualization of phenotypic fingerprint. (A) The principal component analysis and feature importance selection. (a) Screen plot of the PCA model and the curves of cumulative explained variance. (b) Sorting about feature importance. (B) Phenotype ID visualization.

The identified phenotypic traits exhibit substantial physiological and ecological significance, as revealed by the correlation analysis. They play a crucial role in determining lettuce quality and yield. Moreover, these traits demonstrate strong discriminatory potential for varietal classification, particularly in the rapid identification of genetic subgroups. To improve the efficiency and discriminative power of phenotypic feature representation, PCA (Principal Component Analysis) was employed to perform dimensionality reduction on the 17 extracted traits. As shown in Fig. 7A(a), the first 10 principal components accounted for a cumulative contribution of 95.82%. The importance ranking of the features is presented in Fig. 7A(b), and based on this, the top 13 features were selected as the core traits for phenotypic fingerprint construction. These selected features were further standardized and sequentially encoded to form a unique and highly discriminative phenotypic ID, as illustrated in Fig. 7B. This phenotypic ID integrates three dimensions of information shape, color, and texture, and provides an intuitive visualization of the phenotypic diversity among different lettuce varieties in a multidimensional feature space. This greatly enhances the interpretability and visualization of phenotypic data, and provides an innovative technical route and theoretical basis for the precise identification of varieties, germplasm resource management and standardized expression of phenotypic big data.

### 3.4 Subgroup variety identification results

**Fig. 8:**
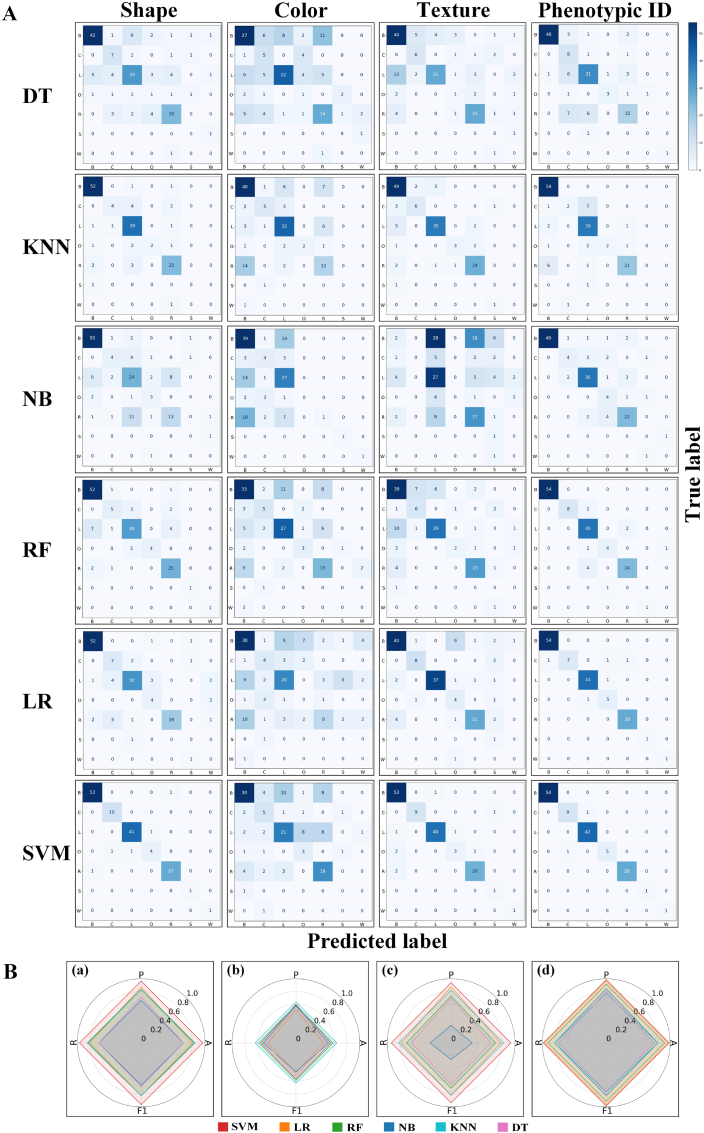
Subgroup variety identification performance. (A) Confusion matrix of subgroup variety identification. (B) Comparison of subgroup variety identification performance. (a) Shape, (b) Color, (c) Texture, (d) Phenotypic ID.

The classification performance of subpopulation varieties was comprehensively evaluated using the constructed phenotypic ID. To benchmark their effectiveness, we also conducted comparative experiments using three types of unidimensional features: shape, color, and texture. The dataset was split into training and testing sets in an 8:2 ratio. Fig. 8A presents the confusion matrices generated from different feature dimensions, highlighting the differences in classification accuracy and misclassification among subgroups. Fig. 8B compares the prediction performance across classifiers. Although unidimensional features perform well in certain models, the phenotypic ID substantially improves accuracy, precision, recall, and F1-score across all algorithms. Among these, the SVM model achieved the best performance, reaching 98.59% accuracy, 98.61% precision, 98.59% recall, and 98.57% F1-score. The multidimensional phenotypic ID integrates shape, color, and texture information, offering a more comprehensive representation of varietal phenotypic variation. Classification models built on this phenotypic ID demonstrate strong discriminative ability and generalization performance, providing a stable and scalable solution for subgroup identification and precise variety classification.

To further examine the effect of hyperparameter optimization, Fig. S2 compares the performance of all classifiers before and after Optuna tuning. The results show that hyperparameter optimization yields clear and consistent improvements across all models in terms of Accuracy, Precision, Recall, and F1-score. Among the classifiers, SVM exhibits the most substantial performance gain and ultimately achieves the highest accuracy. These findings indicate that integrating Optuna-based hyperparameter optimization with the multidimensional phenotypic ID provides a highly reliable and robust framework for lettuce subgroup identification.

## 4 Discussion

### 4.1 Model performance and robustness considerations

Previous semantic segmentation studies have often relied on multi-branch architectures or multi-scale feature fusion schemes to enhance feature representation [43,44], but both strategies tend to struggle with balancing accuracy and computational efficiency. In contrast, LGASSNet adopts a lightweight group attention mechanism that captures multi-scale information within a streamlined backbone. This design avoids the computational redundancy typically seen in multi-branch models and improves fine-detail representation compared with multi-scale fusion approaches. It achieves a favorable balance between accuracy and efficiency and shows particularly strong performance in complex regions such as the main vein, lateral veins, and serrated margins, providing a reliable foundation for precise downstream phenotypic extraction. When combined with the multidimensional phenotypic ID, the framework also achieves high accuracy and stability in subgroup classification, showing reliable discriminative ability even under challenging class boundaries. Although all samples were collected in a controlled greenhouse environment, the group attention mechanisms allow LGASSNet to capture both local details and global structural patterns. This design theoretically provides some potential for inference adaptability across different environments. Meanwhile, controlled illumination was adopted to ensure stable light intensity and spectral composition. Although natural-light acquisition is more accessible in practice, it introduces substantial fluctuations with time and environmental changes, resulting in non-uniform color responses that cannot be fully corrected even through calibration. In contrast, controlled lighting provides a uniform radiometric environment that enhances reproducibility and supports standardized extraction of color-related DUS traits. Consistently, the application of standardized color calibration further improves the comparability of color-related traits across imaging batches and ensures consistency in color-dependent analyses.

Importantly, the relevance of multi-scale structural perception is not limited to leaf phenotyping. Similar observations have been made in root phenotyping tasks, where segmentation continuity and structural integrity directly influence trait extraction quality. Recent work applying an integrated SegFormer-based root phenotyping toolbox demonstrated that preserving fine branching and topological detail significantly improved trait quantification and QTL mining efficiency [45]. Likewise, imaging-based root architecture analysis further showed that multi-scale structural recognition improves phenotype integrity and downstream biological interpretation, reinforcing the importance of fine structural perception in plant image segmentation [46]. These parallels highlight that leaf venation and root architecture share similar branching-like morphological patterns, and that methods capable of capturing multi-scale structural continuity such as LGASSNet hold promise for broader plant phenotyping applications.

Future work will extend evaluations to field environments, and multiple developmental stages. These assessments will help determine the model's robustness under real-world variability and its ability to consistently capture phenotypic variation under genotype × environment (G × E) interactions.

### 4.2 The interpretability and applied potential of the phenotypic fingerprint

The proposed phenotypic fingerprint provides a structured and interpretable representation of lettuce leaf phenotype. Rather than functioning as a simple stacked feature set [47,48], the fingerprint encodes shape, color, and texture traits into a standardized and dimensionless phenotypic code. This representation does not rely on absolute morphological values, but instead reflects relative, biologically grounded trait descriptors. Each trait corresponds to an explicit biological structure, such as LL, LWr, RN, LS, LTS, LVS, LH, and LV, which makes the fingerprint inherently interpretable and aligned with the observability and stability requirements of DUS testing [27,49]. Correlation analysis among traits further reveals coordinated patterns across morphological, textural, and color dimensions, offering insights into biological differentiation among lettuce subgroups. In practical applications, the phenotypic fingerprint supports not only variety identification but also germplasm evaluation, trait pattern analysis, and elite material screening. Its interpretability and scalability enable reliable characterization of phenotypic diversity within breeding resources and provide a solid foundation for intelligent selection and decision-making in modern breeding programs.

### 4.3 Role of the phenotypic fingerprint in genetic differentiation and DUS standardization

The phenotypic fingerprint carries biological significance that extends beyond variety classification. Key traits in the fingerprint, such as LS, LVS, LH, LV, and AH, are jointly influenced by genetic background, photosynthetic efficiency and physiological responses. Their coordinated variation across lettuce subgroups reveals intrinsic patterns of genetic differentiation and provides a phenotypic perspective that aligns with established evolutionary relationships and ecological adaptation strategies. The fingerprint also integrates the quantitative expression of DUS testing traits for lettuce leaves based on UPOV guidelines. These descriptors, which have traditionally relied on subjective visual scoring, are transformed into objective and repeatable phenotypic ID codes. This conversion improves evaluation consistency and reduces assessor-dependent variation. By standardizing the representation of key morphological traits, the phenotypic fingerprint establishes a practical foundation for automating DUS evaluations and supports faster variety testing with higher reproducibility across institutions. Furthermore, integrating the phenotypic fingerprint with genomic, transcriptomic and metabolomic data would enable the construction of more complete genotype–phenotype interaction networks [50,51]. Such multi-omics integration will facilitate intelligent breeding, variety traceability and precision germplasm selection, and it highlights the broad applicability of phenotypic fingerprints.

### 4.4 Limitations and future perspectives

Although the proposed method exhibits robust performance, several limitations remain. First, the present study focuses on constructing phenotypic fingerprints from detached leaves, and the temporal dynamics of trait expression across developmental stages warrant more comprehensive investigation. Second, although the fingerprint-based approach enables accurate subgroup identification, achieving fine-grained, one-to-one variety-level discrimination remains an important direction for future research.

Future work will therefore focus on multi-environment, multi-stage, and multi-sensor in-situ field measurements. Moreover, integrating genomic and transcriptomic multi-omics information will enable the construction of more comprehensive fingerprints that incorporate morphological, color, texture, physiological, and biochemical traits. These advances will support accurate variety-level identification and further strengthen high-throughput phenotyping for intelligent breeding applications.

## 5 Conclusion

This study proposes a high-throughput phenotypic trait analysis pipeline capable of accurately quantifying key leaf-level phenotypic features of lettuce such as shape, color, and texture from 2D images across multiple dimensions. The quantification results revealed significant inter-varietal differences in multidimensional phenotypic traits, which can be effectively captured and distinguished through the construction of phenotypic fingerprints. Using the derived Phenotypic ID, the identification accuracy for lettuce subpopulation varieties reached 98.59%, offering a viable technological pathway for the rapid identification, precise management, and intelligent breeding of lettuce germplasm resources.

Moving forward, the study will further refine phenotypic fingerprints at both the single plant and population scales. The goal is to develop a high-resolution, multi-scale, and multi-temporal digital phenotypic fingerprint map at the variety level. In addition, the transferability of this approach to major staple crops such as maize, wheat, and soybean will be explored. This will promote the integration of high-throughput phenotyping technologies into germplasm discovery, key trait analysis, and molecular design breeding, accelerating the transformation of modern crop breeding toward digitalization and intelligent decision-making.

## Author contributions

Guangjie Qiu: Writing – review & editing, Writing – original draft, Visualization, Methodology, Conceptualization. Weiliang Wen: Writing – review & editing, Methodology. Xiaoqian Chen: Resources, Formal analysis, Data curation. Chuanyu Wang: Investigation, Data curation. Si Yang: Resources, Conceptualization. Xinyu Guo: Supervision, Funding acquisition. Chunjiang Zhao: Validation, Project administration, Funding acquisition.

## Funding

This research is supported by the National Key R&D Program (2022YFD2002300), Collaborative Innovation Center of Beijing Academy of Agricultural and Forestry Sciences (KJCX20240406), and Postdoctoral fund of Beijing Academy of Agriculture and Forestry Sciences.

## Data availability

The data used to support the findings of this study are available upon request from the corresponding author, and the source code is accessible at https://github.com/qiuguangjie87/PP_Phenotypic_Fingerprint.

## Declaration of competing interest

The authors declare the following financial interests/personal relationships which may be considered as potential competing interests: Given their respective roles as Advisory Board member and Associate Editor, Chunjiang Zhao and Weiliang Wen had no involvement in the peer review of this article and had no access to information regarding its peer review. Full responsibility for the editorial process for this article was delegated to another journal editor.
