# Supplementary material for: Quantification of lettuce leaf DUS test traits and phenotypic fingerprint construction for variety identification
Source: Plant Phenomics. 2026 Mar 6;8(2):100197. doi: 10.1016/j.plaphe.2026.100197 (PMC13316521; doi:10.1016/j.plaphe.2026.100197)
Supplement: Multimedia component 1 [file mmc1.docx]

**Quantification of lettuce leaf DUS test traits and phenotypic fingerprint construction for variety identification**

Guangjie Qiu^1,2,3^, Weiliang Wen^2,3^, Xiaoqian Chen^2,3^, Chuanyu Wang^2,3^, Si Yang^2,3,*^, Xinyu Guo ^2,3,*^, Chunjiang Zhao ^1,2,3,*^

^1^Institute for the Smart Agriculture, Jilin Agricultural University, Changchun 130118, China.

^2^Beijing Key Laboratory of Digital Plant, National Engineering Research Center for Information Technology in Agriculture, Beijing 100097, China.

^3^Information Technology Research Center, Beijing Academy of Agriculture and Forestry Sciences, Beijing 100097, China.

*Corresponding authors. E-mail addresses: yangsi4212@163.com (S. Yang), guoxy73@163.com (X. Guo), zhaocj@nercita.org.cn (C. Zhao).

**Supplementary Material:**


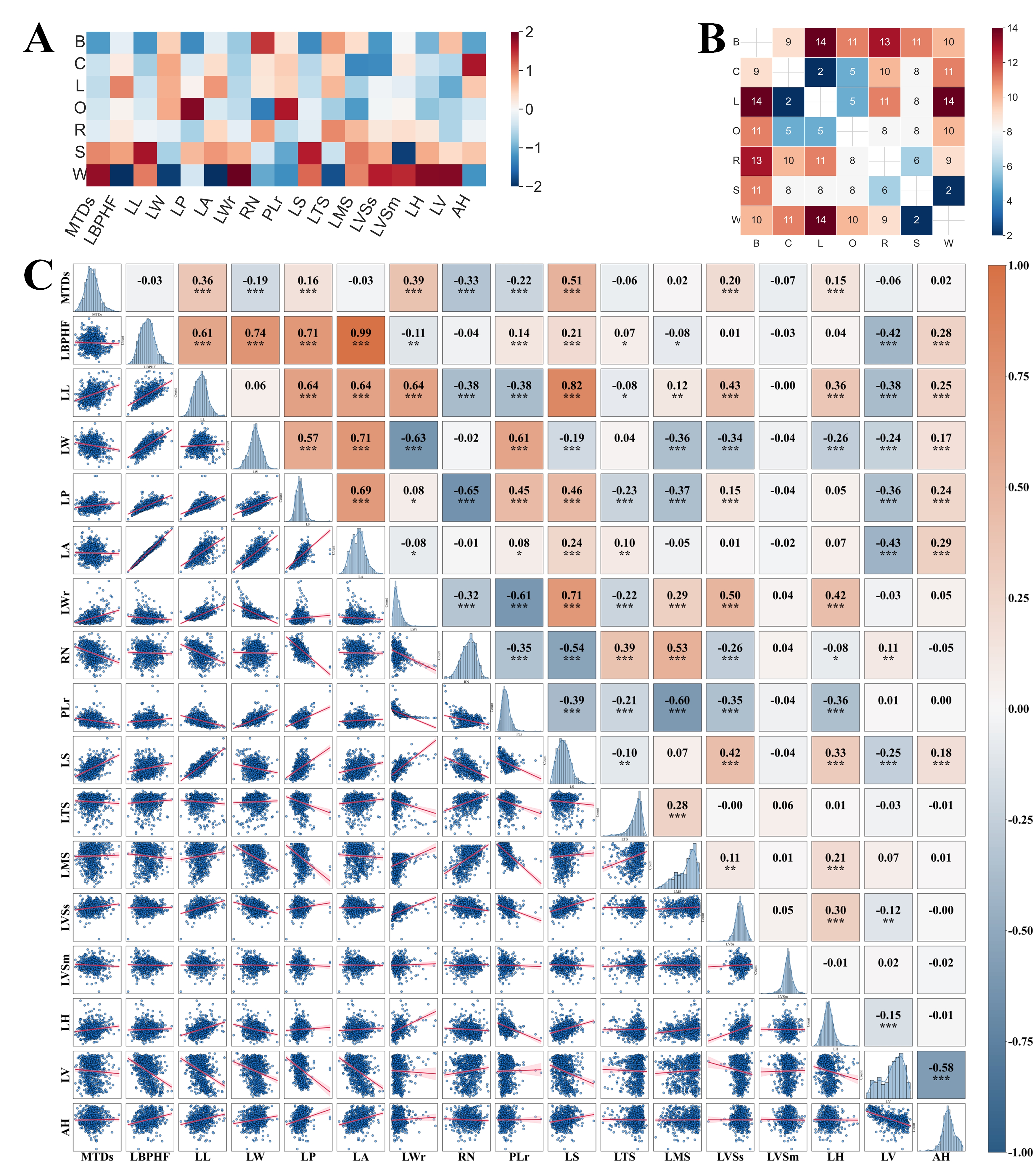


Fig. S1. Statistical analysis of phenotypic trait variation among lettuce subgroups. (A) Heatmap of standardized trait means across seven subgroups. (B) Tukey’s HSD pairwise significance matrix. (C) Pearson correlation analysis of 17 phenotypic traits.


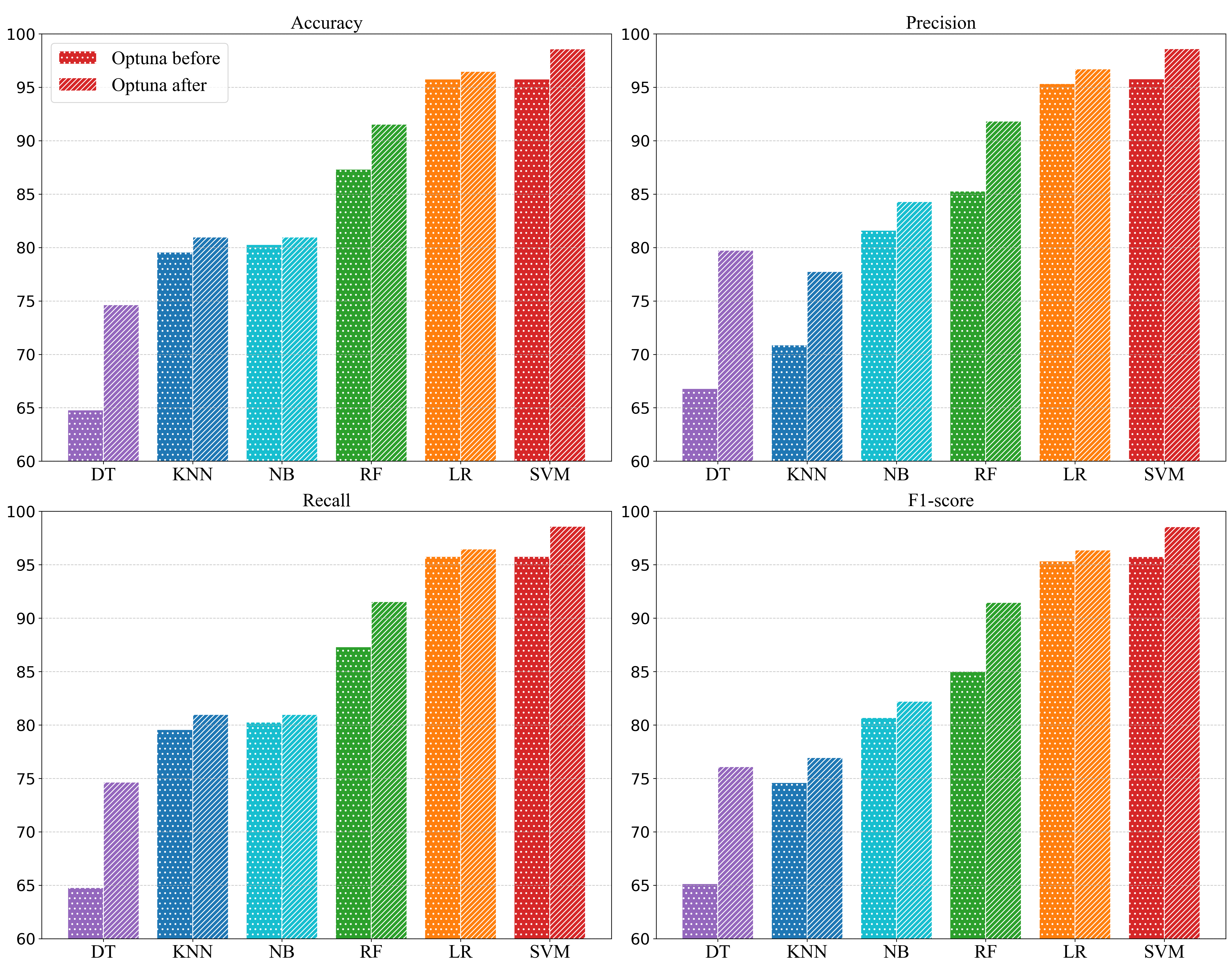


Fig. S2. Performance comparison before and after hyperparameter optimization.

| Table S1. Definition of phenotypic traits in lettuce leaves | | | |
| --- | --- | --- | --- |
| Classification | Trait | Is DUS | Description |
| Shape traits | MTDs | N | multiscale triangle descriptor |
|  | LL | N | leaf length |
|  | LW | N | leaf width |
|  | LP | N | leaf perimeter |
|  | LA | N | leaf area |
|  | LWr | N | leaf length to leaf width ratio |
|  | PLr | N | leaf perimeter to leaf diameter ratio |
|  | RN | N | roundness |
|  | LS | Y | leaf shape |
|  | LTS | Y | leaf tip shape |
|  | LMS | Y | leaf margin shape |
|  | LVS | Y | leaf vein shape |
| Color traits | LH | Y | leaf hue |
|  | LV | Y | color brightness |
|  | AH | Y | Anthocyanin coloration |
| Texture traits | LBPHF | N | LBP-HF texture feature |

| Table S2. Model parameters | |
| --- | --- |
| Parameter | Value |
| Optimizer | Adam |
| Momentum | 0.937 |
| Batch size | 16 |
| Image size | 512×512 |
| Epochs | 400 |
| Learning rate | 5e-4 |

| Table S3. ANOVA for phenotypic traits across lettuce subgroups | | | | |
| --- | --- | --- | --- | --- |
| Trait | F-value | p-value | −log10(p) | Significance |
| MTDs | 14.97 | 3.74 × 10^-16^ | 15.43 | *** |
| LBPHF | 9.03 | 1.59 × 10^-9^ | 8.80 | *** |
| LL | 66.93 | 9.20 × 10^-66^ | 65.04 | *** |
| LW | 33.46 | 1.42 × 10^-35^ | 34.85 | *** |
| LP | 60.45 | 2.36 × 10^-60^ | 59.63 | *** |
| LA | 9.25 | 8.94 × 10^-10^ | 9.05 | *** |
| LWr | 183.44 | 4.03 × 10^-140^ | 139.39 | *** |
| RN | 113.85 | 3.84 × 10^-100^ | 99.42 | *** |
| PLr | 57.99 | 2.94 × 10^-58^ | 57.53 | *** |
| LS | 72.06 | 6.43 × 10^-70^ | 69.19 | *** |
| LTS | 51.93 | 6.08 × 1^-53^ | 52.22 | *** |
| LMS | 84.78 | 9.18 × 10^-80^ | 79.04 | *** |
| LVSs | 33.01 | 3.98 × 10^-35^ | 34.4 | *** |
| LVSm | 1.77 | 1.02 × 10^-1^ | 0.99 | ns |
| LH | 29.03 | 4.08 × 10^-31^ | 30.39 | *** |
| LV | 15.44 | 1.12 × 10^-16^ | 15.95 | *** |
| AH | 2.24 | 3.75 × 10^-2^ | 1.43 | * |

ns p ≥ 0.05; * p < 0.05; ** p < 0.01; *** p < 0.001.
